# Multiple pathways facilitate the biogenesis of mammalian tail-anchored proteins

**DOI:** 10.1242/jcs.207829

**Published:** 2017-11-15

**Authors:** Joseph Casson, Michael McKenna, Sarah Haßdenteufel, Naama Aviram, Richard Zimmerman, Stephen High

**Affiliations:** 1 Division of Molecular and Cellular Function, School of Biological Sciences, Faculty of Biology, Medicine and Health, University of Manchester, Manchester Academic Health Science Centre, Michael Smith Building, Manchester, M13 9PT, UK; 2 Department of Medical Biochemistry and Molecular Biology, Saarland University, 66421 Homburg, Germany; 3 Department of Molecular Genetics, Weizmann Institute of Science, Rehovot 7610001, Israel

**Keywords:** Endoplasmic reticulum, Membrane protein, Protein translocation, SND, SRP

## Abstract

Tail-anchored (TA) proteins are transmembrane proteins with a single C-terminal transmembrane domain, which functions as both their subcellular targeting signal and membrane anchor. We show that knockout of TRC40 in cultured human cells has a relatively minor effect on endogenous TA proteins, despite their apparent reliance on this pathway *in vitro*. These findings support recent evidence that the canonical TRC40 pathway is not essential for TA protein biogenesis *in vivo*. We therefore investigated the possibility that other ER-targeting routes can complement the TRC40 pathway and identified roles for both the SRP pathway and the recently described mammalian SND pathway in TA protein biogenesis. We conclude that, although TRC40 normally plays an important role in TA protein biogenesis, it is not essential, and speculate that alternative pathways for TA protein biogenesis, including those identified in this study, contribute to the redundancy of the TRC40 pathway.

## Abstract (precis)

**Summary:** In addition to the canonical TRC40-targeting pathway, mammalian tail-anchored proteins can also utilise the SRP and SND pathways to facilitate their insertion into the ER membrane.

## INTRODUCTION

Targeting of membrane and secretory proteins to the mammalian endoplasmic reticulum (ER) can occur through either a co-translational SRP-dependent pathway (Nyathi et al., 2013) or distinct post-translational pathways (Johnson et al., 2013). In both cases, the binding of specific cytosolic targeting factors to hydrophobic signal sequences or transmembrane domains prevents their exposure and thereby minimises subsequent inappropriate interactions that might lead to their aggregation (Cross et al., 2009).

An estimated 3–5% of membrane proteins have a C-terminal tail anchor and many of these tail-anchored (TA) proteins are essential for key cellular processes, including membrane fusion and vesicle trafficking (Hegde and Keenan, 2011). The mammalian cytosolic ATPase TRC40 (also known as ASNA1 and Get3) was first identified as an ER-targeting factor that binds post-translationally to the C-terminal hydrophobic domains of TA proteins and facilitates their delivery to the ER, once they have been released from the ribosome (Favaloro et al., 2008; Stefanovic and Hegde, 2007). The post-translational delivery of TA proteins by TRC40 is normally facilitated by two upstream factors, SGTA (Chartron et al., 2012; Leznicki et al., 2011; Mock et al., 2015) and the heterotrimeric BAG6 complex (BAG6, TRC35 and UBL4A) (Leznicki et al., 2010; Mariappan et al., 2010), which act in concert to enable substrate transfer to TRC40 (Casson et al., 2016; Shao et al., 2017). Once a TA protein cargo is loaded onto TRC40, a heterodimeric ER membrane receptor complex (WRB–CAML) facilitates its insertion (Vilardi et al., 2011; Wang et al., 2014; Yamamoto and Sakisaka, 2012).

Although TRC40 is proposed to be the canonical soluble factor that facilitates the post-translational delivery of TA proteins to the mammalian ER, the characterisation of this component has mainly relied upon the use of cell-free approaches (Favaloro et al., 2008; Rabu et al., 2008; Stefanovic and Hegde, 2007). Furthermore, whilst a TRC40 knockout is embryonic lethal in mice (Mukhopadhyay et al., 2006) and disruption of the gene encoding the CAML subunit prevents embryonic development (Tran et al., 2003), loss of the WRB receptor subunit affects only a small number of TA proteins and results in comparatively modest phenotypes in both zebrafish and tissue-specific mouse knockout models (Daniele et al., 2016; Lin et al., 2016; Rivera-Monroy et al., 2016; Vogl et al., 2016). Together, these findings support the hypothesis that the TRC40-mediated post-translational delivery of TA proteins to the ER is not essential *in vivo* and raise the possibility that other ER-targeting pathways may compensate in its absence.

One such alternative was first proposed by Abell et al. (2004), who showed by chemical crosslinking that the signal recognition particle (SRP) can associate with TA proteins and facilitate their SRP receptor (SR)-dependent membrane integration *in vitro*. As the synthesis of tail-anchored proteins must be terminated whilst their ER-targeting signals are still inside the ribosomal exit tunnel (Kutay et al., 1993), and SRP normally binds to these hydrophobic domains during translation (Walter et al., 1981), it was suggested that SRP may bind to TA proteins in a post-translational, but ribosome-dependent manner (Abell et al., 2004; Berndt et al., 2009).

More recently, a third conserved ER-targeting pathway has emerged from studies in yeast (Aviram et al., 2016) and in a human cell line (Haßdenteufel et al., 2017). This SRP-independent (SND)-targeting pathway can accommodate a range of membrane proteins, particularly those that are not fully dependent on either the SRP or GET pathways (Aviram et al., 2016; Haßdenteufel et al., 2017). In yeast, the SND pathway is composed of three components: ribosome-associated Snd1, and two ER transmembrane proteins (Snd2 and Snd3) that form a complex with the Sec61 translocon, and thereby facilitate membrane insertion (Aviram et al., 2016). The mammalian orthologue of yeast Snd2, hSnd2 (also known as TMEM208), has recently been shown to provide an alternative ER-delivery route for proteins with a C-terminal transmembrane domain (Haßdenteufel et al., 2017).

In this study, we show that TRC40 is dispensable for the ER insertion of model TA proteins both in cells and *in vitro*, consistent with the suggestion that this pathway may not be essential for TA protein biogenesis (Rivera-Monroy et al., 2016). Furthermore, our data indicate that the previously observed binding of SRP to TA proteins (Abell et al., 2004; Leznicki et al., 2010) reflects a functional ER-delivery pathway. We also provide experimental evidence for a functional SND pathway in mammals by confirming a role for hSnd2 in the biogenesis of TA proteins (see also Haßdenteufel et al., 2017). The effects of combining the perturbation of different pathways in our cell-based model suggests that additional complexity underlies TA protein biogenesis, and we conclude that multiple alternative pathways can facilitate their ER insertion. These findings begin to provide a molecular basis for the apparent redundancy of the TRC40 pathway, and suggest that additional mechanisms for ER delivery may still await discovery.

## RESULTS

### Deletion of TRC40 has differential effects on endogenous TA proteins

**Fig. 1.**
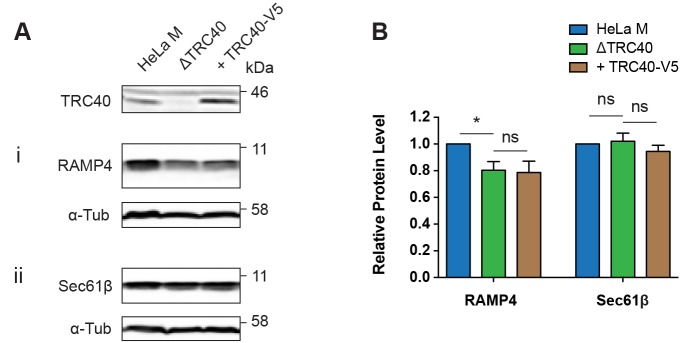
**Deletion of TRC40 has differential effects on endogenous TA protein levels.** (A) The indicated tail-anchored (TA) proteins were analysed by immunoblotting extracts from HeLa M cells and ΔTRC40 cells transfected with pcDNA5 as a vector control, and ΔTRC40 cells rescued by TRC40-V5 transfection. (B) Quantification of TA protein steady-state levels from A relative to α-tubulin (α-Tub) as a cell lysate loading control. HeLa M sample quantifications from each immunoblot were analysed as the control and other conditions were calculated relative to the HeLa M sample in each biological repeat. Data shown are mean±s.d. (*n*=3 biological replicates) and asterisk indicates the significance level between samples shown with a horizontal line. *P≤0.05; ns, not significant (two-way ANOVA).

Previous studies of the contribution of the TRC40 pathway to TA protein biogenesis in metazoans have relied upon the perturbation of the WRB–CAML receptor complex (Daniele et al., 2016; Lin et al., 2016; Rivera-Monroy et al., 2016; Vogl et al., 2016). In order to better understand the role of the soluble TRC40 component in the post-translational delivery of TA proteins to this ER membrane receptor complex, we generated a CRISPR/Cas9-mediated TRC40 knockout in the HeLa M cell line (Fig. 1A and Fig. S1A, see ΔTRC40).

In order to determine the effect of the TRC40 knockout on TA protein biogenesis, we analysed the steady-state levels of three endogenous TA proteins, all of which have previously been suggested to utilise the TRC40 pathway (Favaloro et al., 2008, 2010; Rivera-Monroy et al., 2016). Quantitative immunoblot analysis of total cell lysate revealed that the TRC40 knockout had differential effects on the steady-state levels of these three TA proteins, resulting in a significant reduction of RAMP4 (Fig. 1Ai,B) and STX5 (both isoforms) (Fig. S1Bi,ii), but no effect on Sec61β levels (Fig. 1Aii,B).

In the case of STX5, we also observed a subtle change in its subcellular localisation in the absence of TRC40 (Fig. S1C), as previously reported following the tissue-specific knockout of WRB in mouse cardiomyocytes and hepatocytes (Rivera-Monroy et al., 2016). In contrast, the subcellular distribution of Sec61β showed no obvious perturbation in the ΔTRC40 cell line (Fig. S1D). Both the decrease in STX5 levels and the perturbation of its subcellular distribution could be partially reversed by the exogenous expression of TRC40-V5 (Fig. S1B,C), whereas the reduction in RAMP4 levels was not affected by TRC40-V5 expression (Fig. 1A,B). Taken together, these data support the proposal that the role of the TRC40 pathway for TA protein biogenesis is both precursor specific and partially redundant at a cellular level (Rivera-Monroy et al., 2016).

### TRC40 is not essential for the translocation of TA proteins *in vitro*

To further assess the role of the TRC40 pathway in the post-translational delivery of TA proteins to the ER, we used a well-established *in vitro* system that allows the study of membrane insertion into ER-derived microsomes in the absence of ongoing protein synthesis (cf. McKenna et al., 2016). On the basis of our cell-based studies, we chose RAMP4 for further investigation since we had found it to exhibit some level of TRC40 dependency in cells (Fig. 1A,B), together with Sec61β, which was previously shown to utilise TRC40 *in vitro* (Favaloro et al., 2008), but showed no clear dependence on either TRC40 (Fig. 1A,B), or its WRB receptor subunit (Rivera-Monroy et al., 2016) in cells. In addition, we also included the TA protein CytB5, since, although we were unable to detect the endogenous protein by immunoblotting of whole cell lysate (data not shown), it is a well-defined example of a TA protein that is independent of TRC40 with respect to its insertion into the ER membrane *in vitro* (Favaloro et al., 2008; Stefanovic and Hegde, 2007). By including a C-terminal N-glycosylation tag (denoted OPG or OPG2, Fig. S2A,B), we were able to confirm authentic membrane integration of all three model TA proteins into canine pancreatic rough microsomes (RMs) via a well-established, N-linked glycan-dependent size shift (Abell et al., 2007; Favaloro et al., 2008; Leznicki et al., 2011; cf. Fig. S2A).

**Fig. 2.**
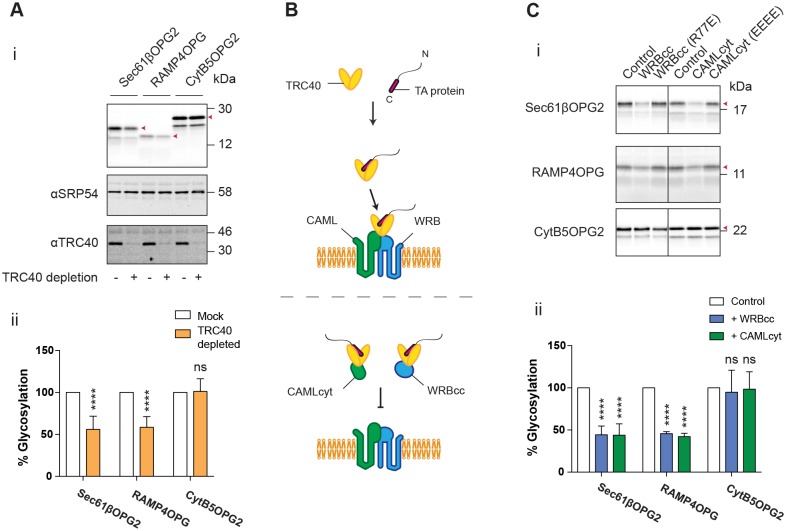
**TRC40 is not essential for post-translational translocation of TA proteins *in vitro*.** (A) Phosphorimage of the indicated TA proteins translated *in vitro* with normal reticulocyte lysate or after TRC40 immunodepletion (i). (ii) Quantification of TA protein N-glycosylation relative to a mock depletion control. (B) Model for the function of CAMLcyt and WRBcc constructs used to inactivate TRC40 in the *in vitro* lysate. (C) Phosphorimage of the indicated TA proteins translated *in vitro*, either in control lysate or after addition of WRBcc or CAMLcyt (i). (ii) Quantification of TA protein N-glycosylation relative to the lysate control with the addition of an equal volume of buffer only. Red arrowheads indicate N-glycosylated species. Quantifications shown are mean±s.d. (*n*=3 technical replicates) and asterisks indicate significance level, compared with control conditions. ****P≤0.001; ns, not significant (two-way ANOVA).

We first tested the membrane integration of these three model TA proteins in a strictly post-translational assay, i.e. where RMs are added to the reaction only after *in vitro* protein synthesis has been terminated and newly synthesised polypeptides have been released from the ribosome by treatment with puromycin (McKenna et al., 2016). To assess its role in TA protein integration, we immunodepleted TRC40 from the translation lysate (Johnson et al., 2012; Leznicki et al., 2010) and examined the membrane insertion of TA protein substrates. After isolation of the resulting membrane fractions, we observed an ∼50% reduction in the membrane insertion of both Sec61βOPG2 and RAMP4OPG (Fig. 2Ai,ii). In contrast, no loss of CytB5OPG2 integration was observed (Fig. 2Ai,ii), consistent with previous studies demonstrating that this TA protein integrates into the ER membrane independently of TRC40 (Favaloro et al., 2008; Stefanovic and Hegde, 2007).

As an alternative method of perturbing the TRC40 pathway, we incubated newly synthesised TA proteins with an excess of the cytosolic domain of the WRB receptor (WRBcc), which competitively blocks endogenous TRC40, such that TA proteins which use this pathway will be prevented from reaching the ER membrane (Vilardi et al., 2011; Fig. 2B). As a control, we generated a second version of WRBcc containing a point mutation that prevents its interaction with TRC40 [WRBcc(R77E); Fig. S2C]. In the presence of WRBcc, we again observed a ∼50% reduction in both Sec61βOPG2 and RAMP4OPG integration into RMs, but found no effect on CytB5OPG2 integration (Fig. 2Ci,ii). Furthermore, this effect was not observed with the WRBcc(R77E) mutant. Similar results were also observed using the cytosolic domain of the CAML receptor (CAMLcyt) and its respective TRC40-binding-deficient mutant, CAMLcyt(EEEE) (Fig. 2Ci,ii).

We speculated that the population of the two ‘TRC40-dependent’ TA proteins, RAMP4 and Sec61β, that was still capable of successful membrane integration in these TRC40 pathway-perturbed systems might be explained by the activity of a small fraction of TRC40 that was either not immunodepleted or that had not been competitively bound by recombinant WRBcc or CAMLcyt. However, no additional reduction in TA protein integration could be achieved when either of the inhibitory WRBcc or CAMLcyt fragments was combined with a TRC40-depleted lysate (Fig. S2D). We therefore conclude that membrane insertion via residual TRC40 is unlikely in this system. Importantly, a proportion of untagged Sec61β was also still capable of successful ER integration following TRC40 depletion (Fig. S2E), indicating that our observations are not simply a consequence of the C-terminal OPG or OPG2 extensions that were added to our model TA substrates (Fig. S2A,B). On the basis of these experiments, we conclude that there are compensatory or redundant pathways that are capable of delivering TA proteins to the ER membrane, even in the absence of a functional TRC40 pathway.

### The membrane components SRα and hSnd2 promote the biogenesis of TA proteins

The redundancy of the TRC40 pathway in the context of TA protein biogenesis suggested that other pathways might be capable of compensating for the lack of TRC40 in our knockout cells. Two alternative ER-targeting pathways seemed to be the most likely candidates for mediating any such alternative route(s) for TA protein delivery: the SRP pathway (Abell et al., 2004) and the recently discovered SND pathway (Aviram et al., 2016; Haßdenteufel et al., 2017).

**Fig. 3.**
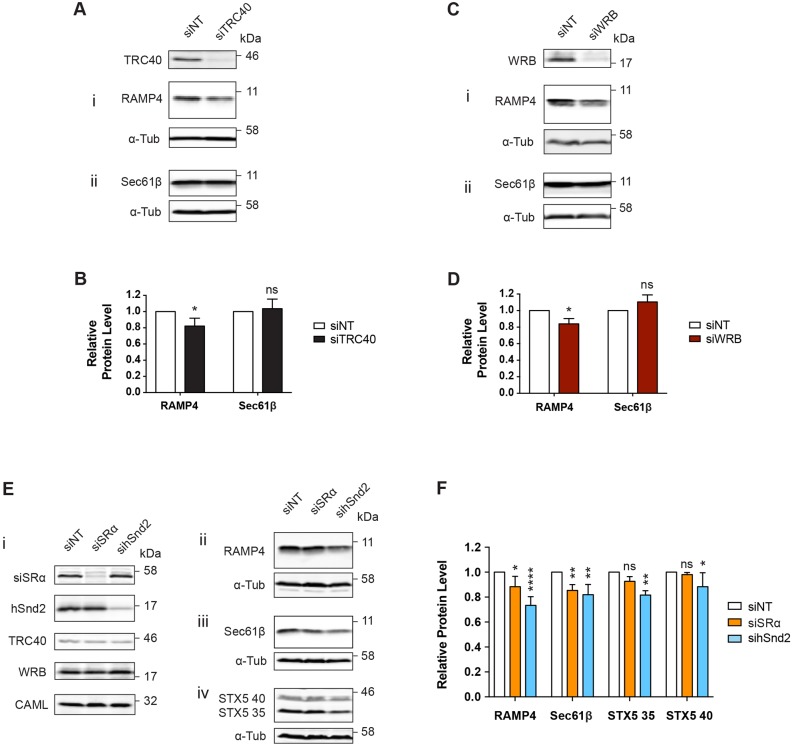
**The membrane components SRα and hSnd2 promote the biogenesis of TA proteins.** (A,C,E) HeLa M cells treated with the indicated siRNAs for 48 h prior to lysate preparation. Samples were analysed for the proteins indicated by immunoblotting. (B,D,F) Quantification of TA protein steady-state levels from A,C,E, respectively, relative to α-tubulin (α-Tub) as a cell lysate loading control; all samples were compared to the non-targeting siRNA control. Data shown are mean±s.d. (*n*=3 biological replicates) and asterisks indicate the significance level of conditions compared with a non-targeted siRNA control. *P≤0.05; **P≤0.01; ****P≤0.001; ns, not significant (two-way ANOVA).

To directly compare the effects of perturbing these three distinct pathways, we used siRNA-mediated depletion of pathway components. We first benchmarked this approach by comparing the effects of a TRC40 knockdown on endogenous TA protein levels (siTRC40) with those following a complete TRC40 knockout. As observed with the ΔTRC40 cell line (Fig. 1A,B), efficient knockdown of TRC40 led to a modest but significant reduction in the steady-state level of endogenous RAMP4, but showed no effect on Sec61β (Fig. 3A,B). Likewise, depletion of the ER membrane receptor component, WRB, resulted in a reduction in the level of RAMP4, whereas no effect on Sec61β was observed (Fig. 3C,D). We therefore proceeded with siRNA-mediated knockdowns of specific components from other ER-delivery pathways, in an effort to determine their respective contributions to TA protein biogenesis.

We compared the effects of knocking down the α-subunit of the SRP receptor (SRα, also known as SRPRA) and hSnd2 on the steady-state levels of endogenous TA proteins. Consistent with an earlier *in vitro* study (Abell et al., 2004), the siRNA-mediated knockdown of SRα resulted in a modest reduction of both RAMP4 and Sec61β levels (Fig. 3E,F). Strikingly, STX5, a substrate that appears to be more sensitive to perturbation of the TRC40 pathway (Fig. 1A,B; Rivera-Monroy et al., 2016) was not significantly affected by depletion of SRα (Fig. 3Eiv,F), whereas, the knockdown of hSnd2 resulted in a significant reduction in the endogenous levels of RAMP4, Sec61β and STX5 (Fig. 3E,F).

In a recent study (Haßdenteufel et al., 2017), it was found that knockdown of hSnd2 in HeLa cells resulted in elevation of the mRNA levels for both subunits of the SRP receptor (SRα and SRβ). Similarly, the depletion of SRα or WRB led to increased levels of the hSnd2 protein, whilst depletion of hSnd2 resulted in an increase in SRα levels (Haßdenteufel et al., 2017). In contrast, our qualitative immunoblot analysis of HeLa M cells showed no obvious compensatory changes in the protein levels of SRα, hSnd2, TRC40, WRB and CAML, following the depletion of SRα or hSnd2 in whole cell extracts (Fig. 3Ei). Likewise, membrane-enriched fractions prepared by digitonin permeabilization (cf. Wilson et al., 1995) showed no indication of such changes in alternative ER receptor subunits (Fig. S3A). Nevertheless, it is possible that depletion of hSnd2 in HeLa M cells results in subtle changes to the levels of these components that were not apparent from our analysis. In any case, it would appear that like TA proteins, the subunits of the ER membrane receptors analysed in this study can exploit multiple pathways for ER integration. Taken together, these findings suggest that at least three pathways contribute to maintaining the endogenous levels of TA proteins and raise the possibility that both the SRP and SND pathways are directly involved in TA protein delivery to the mammalian ER.

### TA proteins can utilise the SRP pathway post-translationally

**Fig. 4.**
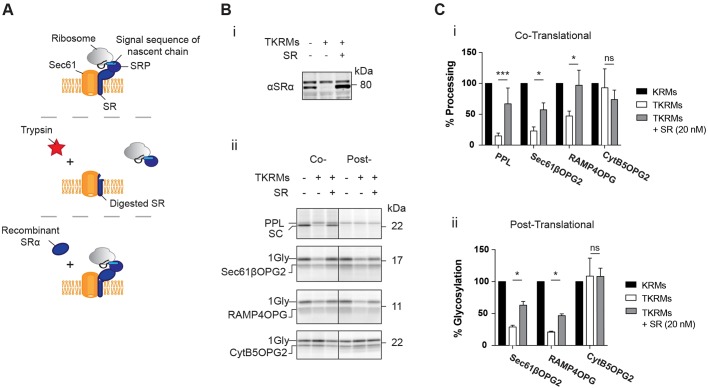
**TA proteins can utilise SRP post-translationally.** (A) Model for the trypsinisation and re-addition of recombinant SR (SRα and SRβ lacking its transmembrane domain, Jadhav et al., 2015) to rescue SR function. (B) Phosphorimage of (i) the SR rescue experiment and (ii) the indicated TA proteins that were *in vitro* translated in the presence of control salt-washed rough microsomes (KRMs) or KRMs after trypsinisation (TKRMs) and subsequent SR re-addition (SR). TA protein N-glycosylation (Gly) or signal cleavage (SC) levels were normalised to lysate control in the co-translational (i) and post-translational systems (ii). Data shown are mean±s.d. (*n*=3 technical replicates) and asterisks indicate the significance level between samples shown with a horizontal line. *P≤0.05; ***P≤0.005; ns, not significant (two-way ANOVA).

Our finding that perturbation of the SRP receptor complex in cells had an effect on the steady-state levels of two endogenous TA proteins (Fig. 3E,F), together with previous *in vitro* studies suggesting that some TA proteins may interact productively with SRP in a post-translational manner (Abell et al., 2004; Leznicki et al., 2010) led us to speculate that the SRP pathway might be able to play a significant role in TA protein biogenesis. To further address the potential role of the SRP pathway in the post-translational ER delivery of TA proteins, we performed *in vitro* integration assays using trypsinised RMs. Whilst such trypsinisation degrades several membrane components, any reduction in ER integration or translocation that is due to the loss of SR can be partially rescued by the addition of intact recombinant SR (Abell et al., 2004; Fig. 4A,Bi).

After analysis of the membrane fractions isolated from these experiments, we found that the translocation of preprolactin (PPL), a model co-translational substrate, is strongly perturbed following trypsinisation. However, as previously reported, this effect is largely reversed upon the addition of exogenous SR (Fig. 4Bii,Ci; cf. Jadhav et al., 2015). The same trend of loss and rescue is observed for the integration of Sec61βOPG2 and RAMP4OPG when they are translated in the presence of ER microsomes (co-translational), but also when these membranes are added after translation is terminated and the substrate is released from the ribosome (post-translational) (Fig. 4B,C). However, no translocation for PPL is observed in this post-translational system (Fig. 4Bii), confirming that the use of the SRP-dependent pathway by Sec61βOPG2 and RAMP4OPG is post-translational. In contrast to the assisted membrane insertion of Sec61βOPG2 and RAMP4OPG, membrane insertion of CytB5OPG2 is unperturbed by any of the manipulations that we performed *in vitro* (Fig. 4B,C).

Furthermore, as previously established for the effect of TRC40 depletion (Fig. S2E), we find that SR-dependent rescue of ER integration with trypsinised RMs is still observed using untagged Sec61β that lacks a C-terminal extension (Fig. S3B). The addition of recombinant SR to non-trypsinised RMs had no effect on either PPL translocation or TA protein integration (Fig. S3C), demonstrating that its addition does not simply enhance translocation non-specifically (Fig. 4B,C). Together, our observations support the previous findings of Abell and colleagues (Abell et al., 2004), and we conclude that the SRP-dependent pathway for ER delivery can promote TA protein biogenesis in cells via a post-translational mechanism.

### Multiple pathways facilitate the insertion of TA proteins in cells

**Fig. 5.**
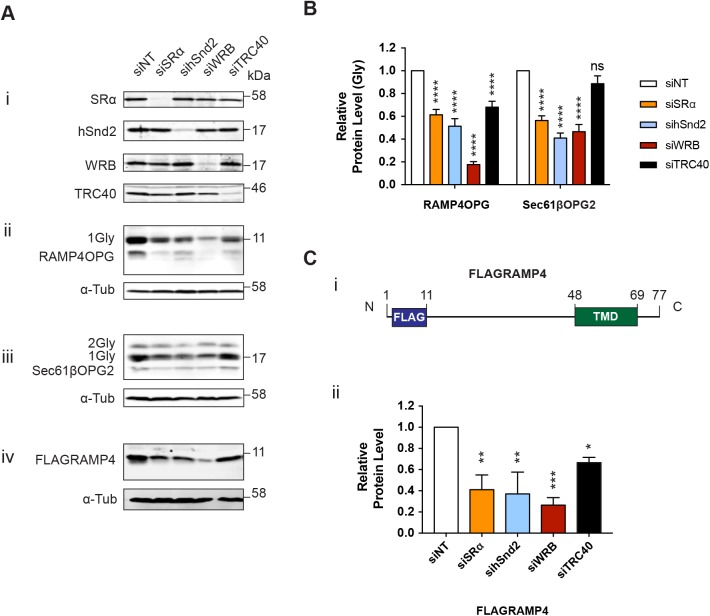
**Multiple pathways facilitate the insertion of TA proteins in cells.** (A) HeLa M cells were treated with the indicated siRNAs for 48 h (i) and transiently transfected with RAMP4OPG (ii), Sec61βOPG2 (iii) or FLAGRAMP4 (iv), for 24 h prior to lysate preparation. Samples were analysed for the proteins indicated by immunoblotting. (B) Quantification of exogenous TA protein N-glycosylation after siRNA knockdown was calculated relative to the non-targeting siRNA control. Significance is shown as ****P≤0.001 (two-way ANOVA). (C) (i) Schematic of FLAGRAMP construct used in A (iv). (ii) Quantification of exogenous FLAGRAMP levels after siRNA knockdown. Data shown are mean±s.d. (*n*=3 biological replicates) and asterisks indicate the significance level of conditions compared with a non-targeted siRNA control. Significance is shown as *P≤0.05; **P≤0.01; ***P≤0.005 (one-way ANOVA).

Having obtained evidence that these alternative pathways can facilitate the biogenesis of TA proteins, we studied the effects of manipulating these different pathways in live cells using exogenous versions of RAMP4 and Sec61β with C-terminal OPG/OPG2 tags (Fig. S2B). In this way, we were able to unambiguously identify a bona fide pool of membrane-inserted, N-glycosylated TA proteins (see Fig. S4A), rather than the potentially mixed population of inserted and non-inserted polypeptides that the endogenous forms of these two TA proteins might represent (Fig. S4A; cf. Figs 1 and 3). Knockdown of a receptor component from each of the three pathways that we had already tested (siSRα, sihSnd2 and siWRB, Fig. 5Ai) resulted in a significant reduction in the N-glycosylated, and therefore membrane-inserted, pool of both RAMP4OPG and Sec61βOPG2; with the WRB knockdown having a particularly strong effect on N-glycosylated RAMP4OPG (Fig. 5A,B). Interestingly, depleting each of these components also appeared to reduce the level of non-glycosylated RAMP4OPG, an effect that could be partially reversed by inhibition of the proteasome (Fig. S4B). We speculate that this represents a cytosolic pool of mislocalised TA proteins that is normally targeted for degradation (Hessa et al., 2011).

As the number of residues after a hydrophobic ER-targeting signal plays a key role in the efficiency with which SRP can co-translationally deliver nascent polypeptides to the ER (Watson et al., 2013), it remained possible that the significant effects of SRα and hSnd2 depletion reflected an increased dependency of our modified exogenous TA proteins on these pathways as a consequence of their non-physiological C-terminal extensions. To address this issue, we created an additional variant of RAMP4, with an N-terminal FLAG tag (FLAGRAMP4; Fig. 5Ci), thereby allowing us to detect the steady-state level of an exogenous model TA protein without the need for a C-terminal extension. Importantly, the trend observed with FLAGRAMP4 is directly comparable to RAMP4OPG and the depletion of each of the three ER membrane receptor components significantly reduced the steady-state level of FLAGRAMP4 (Fig. 5Aiv,Cii). We therefore conclude that all three of these ER membrane receptors can facilitate the biogenesis of TA proteins *in vivo*.

**Fig. 6.**
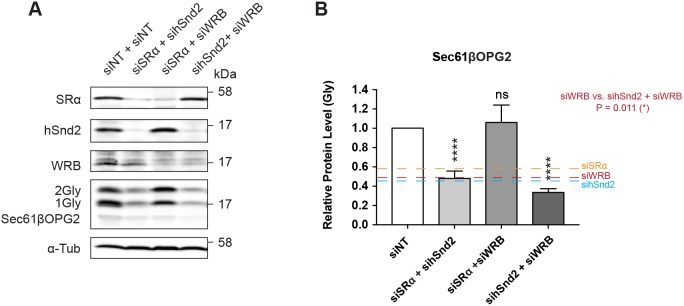
**Co-depletion of ER-delivery pathway components.** (A) HeLa M cells treated with the indicated siRNAs for 48 h and transiently transfected with Sec61β-OPG2, for 24 h prior to lysate preparation. Samples were analysed for the proteins indicated by immunoblotting. (B) Quantification of Sec61βOPG2 N-glycosylation relative to α-tubulin (α-Tub) as a cell lysate loading control. Quantification of exogenous TA protein N-glycosylation after siRNA knockdown relative to the non-targeting siRNA control. Data shown are mean±s.d. (*n*=3 biological replicates) and asterisks indicate the significance level between each condition and the siNT control. ****P≤0.001; ns, not significant; (one-way ANOVA).

To attempt to understand the potential inter-relationship between the different ER-targeting pathways that can accommodate TA proteins, double knockdowns were used to deplete combinations of SRα, hSnd2 and WRB, and the effects on the N-glycosylation of Sec61βOPG2 were compared. A modest additional reduction in the amount of membrane-inserted Sec61βOPG2 was observed following co-depletion of hSnd2 and WRB, compared with the effect of depleting WRB or hSnd2 alone (Fig. 6). In contrast, the effect of depleting both SRα and hSnd2 was directly comparable to depleting hSnd2 alone (Fig. 5B and Fig. 6B).

**Fig. 7.**
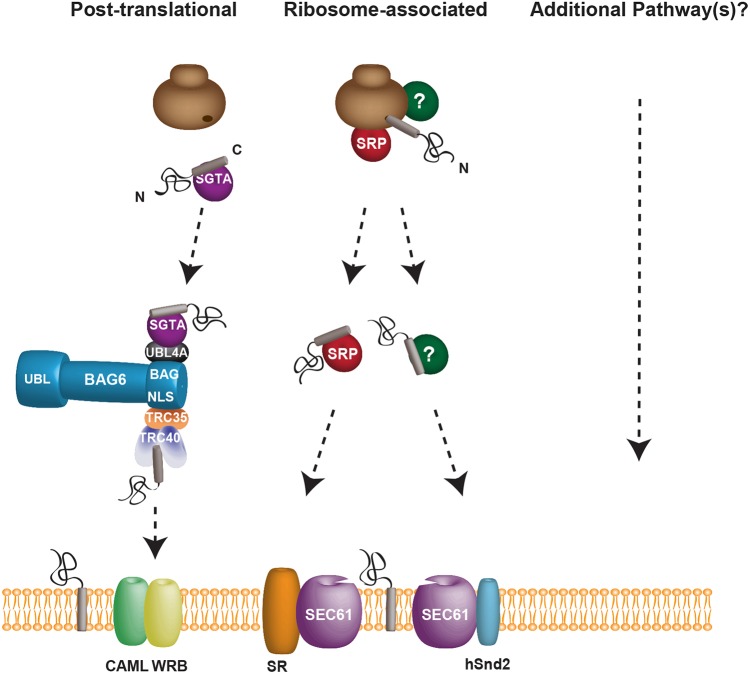
**Multiple pathways facilitate the biogenesis of mammalian tail-anchored proteins.** TA proteins are able to utilise each of the three known ER-delivery pathways to insert into the ER membrane, and our data do not exclude a role for additional, as yet unidentified, routes. The well-characterised TRC40 pathway is not essential for TA protein insertion, and we propose that both the SRP and recently discovered SND pathways contribute to the apparent redundancy of the mechanisms that underlie TA protein biogenesis. A complete understanding of the mammalian SND pathway will require the identification of the mammalian cytosolic factor (labelled ‘?’), which can engage both the hSnd2 membrane receptor and TA proteins, and it is presently unclear whether this pathway acts co- and/or post-translationally (cf. Pool, 2016).

Surprisingly, although depletion of either SRα or WRB had a clear effect on Sec61βOPG2 integration (Fig. 5Aii,B), knockdown of SRα and WRB together reversed these individual effects, resulting in no reduction in Sec61βOPG2 integration compared with control treatment (Fig. 3, Fig. 6). This suggests that under the conditions of this double knockdown, other pathways, probably including the SND pathway, can effectively compensate for the loss of the TRC40 and SRP-mediated routes. Taken together, our data indicate that at least three pathways for ER delivery can each play a significant role in the insertion of mammalian TA proteins into the ER membrane (Fig. 7).

## DISCUSSION

TRC40/Get3-dependent targeting of TA proteins to the ER membrane has been extensively studied and the mechanisms underlying Get3-mediated membrane insertion well characterised (Mateja et al., 2015; Mock et al., 2015; Schuldiner et al., 2008; Shao et al., 2017; Wang et al., 2010). However, the loss of either membrane or soluble components of this pathway in yeast, or a membrane component in mice has a rather modest effect on the steady-state levels of most TA proteins (Jonikas et al., 2009; Rivera-Monroy et al., 2016; Schuldiner et al., 2008). It is therefore apparent that the mammalian TRC40 and yeast GET pathways are not essential for TA protein biogenesis.

In this study, we sought to define the importance of the soluble ER-targeting component TRC40 for TA protein biogenesis and explored the possibility that alternative pathways might also facilitate the ER delivery of TA proteins. We found that both a TRC40 knockout (Fig. 1 and Fig. S1B) and siRNA-mediated TRC40 knockdown (Fig. 3A,B) resulted in distinct effects on different endogenous TA proteins, as demonstrated previously using a tissue-specific WRB knockout mouse model (Rivera-Monroy et al., 2016). Interestingly, on the basis of steady-state protein levels, our data suggest that a knockdown of cytosolic TRC40 has a weaker effect than depletion of the WRB membrane component on the ER insertion of RAMP4OPG and Sec61βOPG2 (Fig. 5A,B). We speculate that the greater impact on TA protein biogenesis observed upon the depletion of a membrane component reflects the fact that TA proteins will more readily enter an alternative ER-targeting route in the absence of an upstream component, such as TRC40. In contrast, once committed to a delivery pathway via an interaction with TRC40, the absence of its cognate ER membrane receptor would most likely result in a ‘dead-end’ scenario. It has also been suggested that in yeast, the Get1–Get2 ER receptor complex facilitates the membrane insertion of TA proteins (Wang et al., 2014) and therefore the loss of WRB could impact on both the delivery to and insertion into the ER membrane.

To focus directly on the role of TRC40 in the post-translational targeting of TA proteins, an *in vitro* translation system was employed. Using TRC40 depletions (Fig. 2A) and dominant-negative WRB/CAML receptor fragments (Fig. 2C), we confirmed that two model TA proteins are still capable of significant membrane insertion (∼50%) in the absence of a functional TRC40 pathway. Interestingly, although perturbation of the TRC40 pathway *in vitro* has similar effects on the membrane insertion of RAMP4OPG and Sec61βOPG2 (Fig. 2A,C), Sec61β shows less dependence on the TRC40 pathway in cells (Figs 3 and 5). It is possible that this difference between *in vitro* and cell-based experiments is due to different proportions of components present *in vitro* compared with cells. Additionally, the strictly post-translational system that was employed *in vitro* may lead to a greater proportion of the TA protein substrates aggregating and/or becoming translocation incompetent (Ngosuwan et al., 2003).

Interestingly, although our TRC40 knockout cells are viable, targeted disruption of the TRC40 gene results in embryonic lethality in mice (Mukhopadhyay et al., 2006). This suggests that under certain conditions, or in particular cell types, TRC40 may be crucial for normal development, perhaps reflecting a requirement for a particularly TRC40-dependent TA protein (cf. Vogl et al., 2016). In yeast, the TRC40 homologue Get3 performs an additional function as an ATP-independent holdase (Voth et al., 2014), and it is also conceivable that TRC40 may play a similar role during mammalian development.

Having shown that TRC40 was not essential for TA protein biogenesis in cultured cells, we hypothesised that the well-defined SRP and/or recently discovered SND pathways might play a role in mammalian TA protein biogenesis (Abell et al., 2004; Aviram et al., 2016; Haßdenteufel et al., 2017). Our *in vitro* data showed that the removal and re-addition of SR significantly affects the efficiency of TA protein insertion into the ER membrane when the process of integration is forced through a strictly post-translational route (Fig. 4Bii and Cii). This is consistent with the model presented by Abell and colleagues, and supports the hypothesis that SRP can engage TA proteins after translation has terminated (Abell et al., 2004). Using a cell culture model, siRNA-mediated depletion of the SRα subunit resulted in a clear reduction in the levels of endogenous RAMP4 and Sec61β (Fig. 3E,F), but had no significant effect on the potentially more TRC40-dependent STX5 (Fig. S1B and Fig. 3E,F; Rivera-Monroy et al., 2016). This lends further support to the idea that SRP can efficiently engage TA proteins and facilitates their membrane insertion via its interaction with the SRP receptor (Abell et al., 2004).

In the case of CytB5, we were unable to compare our OPG-tagged exogenous version of the protein with its endogenous form (cf. Figs 3 and 5). However, during our *in vitro* studies of CytB5OPG2, perturbation of either the TRC40 or SRP-dependent pathways had no effect on its ER insertion (Figs 2 and 4), as previously reported (Abell et al., 2004; Favaloro et al., 2008; Leznicki et al., 2010; Stefanovic and Hegde, 2007). Although we were unable to address the role of hSnd2 using our *in vitro* system, CytB5 integration at the ER is reduced by ∼50% in semi-permeabilised cells that are depleted of both WRB and hSnd2 (Haßdenteufel et al., 2017). However, the knockdown of SRα in this study had a similar result, and the authors concluded that the 28 residue C-terminal extension added to detect the ER integration of CytB5 might contribute to its apparently promiscuous utilisation of different ER-targeting pathways (Haßdenteufel et al., 2017). A definitive understanding of how the human SND pathway plays a role in CytB5 biogenesis will require robust assays for studying the native protein.

In addition to the SRP pathway, we also found evidence that hSnd2, a component of the recently identified human SND pathway (Haßdenteufel et al., 2017), can facilitate TA protein biogenesis in live cells. Strikingly, depletion of hSnd2 resulted in a reduction of the levels of all three endogenous TA proteins tested (RAMP4, Sec61β and STX5) (Fig 3E,F), whereas perturbation of the TRC40 or SRP pathways had more selective impacts on the levels of these TA proteins (Fig. 3). The effects of depleting WRB, SRα and hSnd2 were recapitulated using exogenous (OPG/OPG2-tagged) forms of RAMP4 and Sec61β, which enabled us to confirm that the N-glycosylated and therefore membrane-integrated pool was affected for both TA proteins. Furthermore, in the case of RAMP4, we obtained similar results using an N-terminal epitope tag, indicating that the C-terminal extensions did not fundamentally alter the behaviour of our exogenous TA proteins. These results support the model originally proposed by Aviram and colleagues, in which the SND pathway can compensate for substrates that fail to engage either SRP or Get3 in yeast (Aviram et al., 2016). Our results also support the recent proposal that there is a directly comparable SND pathway in mammals (Haßdenteufel et al., 2017) and demonstrate that in addition to WRB, both SRα and hSnd2 can contribute to the ER integration of TA proteins in cells.

In an effort to understand the relative contributions of these different ER-delivery pathways with respect to TA protein biogenesis, we used a double knockdown approach (cf. Haßdenteufel et al., 2017). Our initial hypothesis was that membrane insertion might be further reduced following the combined perturbation of two potential targeting pathways, and we therefore tested Sec61βOPG2, since no single knockdown resulted in a >50% reduction in its ER integration (Fig. 5B). The only evidence we found for such additive effects of pathway perturbation was a small further reduction upon the depletion of both WRB and hSnd2 (Fig. 6). In contrast, the depletion of both SRα and hSnd2 was no more effective at reducing Sec61βOPG2 integration than knocking down hSnd2 alone (Fig. 6).

Strikingly, the depletion of both SRα and WRB reversed the effects of the individual knockdowns, and the membrane insertion of Sec61βOPG2 was as efficient as under control conditions (Fig. 6). Our understanding of the newly identified human SND pathway is incomplete (Haßdenteufel et al., 2017), and at present, we can only speculate as to the basis for this unexpected outcome. It is possible that the perturbation of both the SRP and TRC40 pathways allows Sec61βOPG2 to be more effectively channelled into the human SND pathway and/or causes an upregulation of SND components (cf. Haßdenteufel et al., 2017), although hSnd2 appears to be unaffected (Fig. 6A). Alternatively, perhaps additional, as yet undefined, ER-targeting pathways are also employed when both the SRP and TRC40 delivery routes are disabled (Fig. 7).

In conclusion, we provide evidence that TA proteins can utilise at least three distinct pathways for their delivery to the ER and our studies leave open the possibility that other routes might also contribute to their biogenesis (Fig. 7). Furthermore, although the SRP-dependent co-translational (Gilmore et al., 1982; Walter et al., 1981) and TRC40-dependent post-translational (Favaloro et al., 2008; Stefanovic and Hegde, 2007) pathways for ER delivery are often considered distinct, the identification of multiple alternative pathways for TA protein biogenesis suggests that any boundaries between different ER-delivery routes may be rather indistinct (Pool, 2016). Consequently, we currently favour a model in which several pathways cater to overlapping repertoires of TA proteins and act in such a way that they may compensate for one another as required (Fig. 7; Aviram et al., 2016; Rivera-Monroy et al., 2016).

## MATERIALS AND METHODS

### Antibodies and DNA constructs

The mouse monoclonal antibody recognising the opsin tag was used as described previously (Adamus et al., 1991). Rabbit antisera against TRC40, RAMP4, Sec61β and SRα were gifts from Bernhard Dobberstein (University of Heidelberg, Heidelberg, Germany). The mouse anti-TRC40 monoclonal antibody was obtained from Abnova (H00000439-M03) and the mouse anti-TAT1 (α-Tub) serum was a gift from Keith Gull (University of Oxford, UK). Rabbit anti-STX5 (110 053), anti-WRB (324 002) and guinea pig anti-CAML (359 004), were all obtained from Synaptic Systems. Rabbit anti-hSnd2 serum was as described in Haßdenteufel et al. (2017). The sheep anti-Golgin 84 antibody was a gift from Martin Lowe (University of Manchester, UK). DNA constructs encoding model TA proteins are as previously described (McKenna et al., 2016), with the exception of FLAGRAMP4 which was generated for this study.

### Cell culture

HeLa M cells used in this study were a gift from Martin Lowe (University of Manchester, UK). Cells were cultured in Dulbecco's modified Eagle's medium (DMEM; Sigma-Aldrich, D6429) supplemented with 10% fetal bovine serum (FBS; Sigma-Aldrich, F9665) and 2 mM L-glutamine (Sigma-Aldrich, G7513). Cells were seeded in sterile 12-well plates (Triple Red, TCP011012) at 7.5×10^4^ cells/ml for immunoblotting analysis and 2.5×10^4^ cells/ml on 13 mm coverslips (SLS, MIC3336) for immunofluorescence microscopy. Plasmid transfections were performed after 24 h of cell growth, using Lipofectamine 2000 transfection reagent and Opti-MEM (Thermo Fisher Scientific, 12566014 and 31985062).

### CRISPR/Cas-9-mediated TRC40 knockout

gRNA sequences used to generate a HeLa M TRC40 knockout cell line (ΔTRC40) were provided in wild-type Cas-9 expressing plasmids (Horizon Discovery). Plasmids containing the gRNA sequences 5′-GTTCGAAGATGCTCCTGATG-3′ and 5′-TTGCTAAGTGTAGGCTCCAG-3′ were co-transfected in HeLa M cells. At 24 h post-transfection, cells were diluted and reseeded in 96-well plates at a dilution of 1 cell/well to produce clonal populations for subsequent validation. Clonal populations of transfected cells were allowed to grow for 2–3 weeks until confluent. Each well of successfully growing cells was tested for the presence of endogenous TRC40 by immunoblotting. Genomic TRC40 DNA of successfully TRC40-depleted cells was isolated using a DNeasy Blood and Tissue Kit (Qiagen, 69504). PCR fragments (∼500 bp) of genomic TRC40 DNA were amplified using primers with the sequences 5′-cggattGGATCCGTAGGGGGAACCCTTGGAAAATTATAGACCAG-3′ and 5′-cggattGAATTCGCTCCAGGTAACTTCCAGAGAGTGAAAC-3′ to add 5′ *Bam*HI-HF and 3′ *Eco*R1-HF restriction sites, for sub-cloning into the pHisTrx vector to allow sequencing of individual chromosome frame shift mutations (GATC Biotech).

### siRNA-mediated knockdown

siRNA oligonucleotides used for knockdown experiments were transfected with INTERFERin (PolyPlus, 409-50), according to the manufacturer's instructions. Sequences of custom oligonucleotides were as follows: sihSnd2, CUAUAGGGUCGUUGAAUAATT (Haßdenteufel et al., 2017), siWRB, AAAUCCAACAGGUAAUUCCAACACC (Rivera-Monroy et al., 2016), siSRα, GAGCUUGAGUCGUGAAGAC (validated internally). The ON-TARGETplus Non-targeting Control siRNA was obtained from Dharmacon (D-001810-0X) and siTRC40 was from Abnova (H00000439-R02). Knockdowns were performed using single rounds of 48 h siRNA transfection in HeLa M cells and subsequent transfections of plasmid DNA encoding proteins of interest were performed 24 h post-siRNA transfection.

### Immunofluorescence microscopy

Cells grown on coverslips were fixed and permeabilised with methanol for 10 min at 4°C. Permeabilised cells were incubated with primary antibodies in PBS (1:100) for 1 h at room temperature, and with appropriate secondary antibodies and DAPI in PBS (1:500 and 1:1000, respectively) for 30 min. Coverslips were mounted onto glass slides and snapshot widefield images were collected on an upright microscope (Olympus BX-60) using a 10×0.30 Plan Fln objective and captured using a Coolsnap ES camera (Roper Scientific) through MetaVue Software (Molecular Devices). The resulting images were processed and analysed using ImageJ and Adobe Illustrator CS4.

### Immunoblotting

Whole cell lysates prepared directly in SDS-PAGE sample buffer, or membrane-enriched fractions prepared by solubilising digitonin-permeabilised cells in sample buffer, were resolved by SDS-PAGE and then transferred onto PVDF membranes (Immobilon, IPFL00010) at 300 mA for 140 min in transfer buffer [25 mM Tris-HCl, pH 8.3, 192 mM glycine, 0.25% (w/v) SDS, 20% (v/v) methanol]. Membranes were incubated in 10% (v/v) blocking buffer (Sigma-Aldrich, B6429) in Tris-buffered saline (TBS) (20 mM Tris-HCl, pH 7.6, 150 mM NaCl) for 1 h at room temperature. Primary antibodies diluted (1:1000) in 10% blocking buffer/TBS-T [TBS with 0.1% (w/v) Tween] were added to membranes overnight at 4°C. Membranes were washed with TBS-T and appropriate secondary antibodies diluted in 10% blocking buffer, TBS-T with 0.01% SDS were added for 1 h. Membranes were washed and scanned using the Odyssey Infrared imaging system (LI-COR Biosciences, 700 nm and 800 nm, 169 μm resolution). Dye-conjugated IRDye secondary antibodies (rabbit or mouse, 680/800 nm; LI-COR) and streptavidin IRDye (LI-COR Biosciences) were used for quantitative immunoblotting (1:10,000).

### *In vitro* translation/translocation assays

*In vitro* translations were performed and ER-derived membranes were recovered as described previously (McKenna et al., 2017). Samples were analysed by phosphorimaging as described previously (McKenna et al., 2016).

### Immunodepletion of TRC40

Protein-A–Sepharose (40 μl bead volume; Genscript, L00210) that had been pre-equilibrated in immunoprecipitation (IP) buffer [10 mM Tris-HCl, pH 7.5, 140 mM NaCl, 1 mM EDTA, 1% (v/v) Triton X-100, 5 mM PMSF and 1 mM L-methionine] was incubated with 65 μl rabbit anti-TRC40 antibody at 4°C on a roller for 2 h. Beads were centrifuged at 13,000* **g*** at 4°C for 1 min and washed three times with buffer R (50 mM HEPES-KOH, pH 7.5, 40 mM potassium acetate, 5 mM MgCl_2_). Nuclease-treated rabbit reticulocyte lysate (RRL; 400 μl) was added to the beads and incubated at 4°C on a roller for 2 h. The mixture was centrifuged at 13,000* **g*** at 4°C for 1 min and the supernatant was collected for translations in immunodepleted lysate. Control mock depletions were carried out in parallel, with an equal volume of water to replace the antibody.

### Expression and purification of WRBcc and CAMLcyt

WRBcc and WRBcc(R77E) were cloned in-frame with an N-terminal thioredoxin (Trx) tag in the pHisTrx vector; CAMLcyt and CAMLcyt(EEEE) were cloned in-frame with an N-terminal glutathione S-transferase (GST) tag in the pGEX-5 vector. These constructs were used to transform BL21 (DE3) pLysS ultra-competent *E. coli* cells for growth and 0.1 M IPTG induction at OD_600_=0.4–0.6 for 3 h at 30°C, followed by centrifugation at 4500* **g*** for 20 min. For Trx-WRBcc constructs, the pellets were each resuspended in 30 ml lysis buffer [50 mM HEPES-KOH, pH 7.5, 300 mM NaCl, 10 mM imidazole, 10% (w/v) glycerol, 5 mM β-mercaptoethanol, 1 mM PMSF and 0.1 mg/ml lysozyme]. For GST–CAML constructs, the pellets were resuspended in lysis buffer without imidazole. Resuspended samples were sonicated for 3×30 s (XL-2000 series sonicator probe; QSONICA), centrifuged at 27,000* **g*** for 30 min at 4°C, and the supernatant was passed through a 0.45 μm filter.

For Trx-WRBcc constructs, 0.5 ml bead volume of Ni-NTA beads (Invitrogen, R901-01) equilibrated in lysis buffer was added to the filtered supernatant on a roller overnight at 4°C and then added to a poly-prep column (Bio-Rad, 7311550). The column was washed with 10 ml of the following buffers: wash 1, lysis buffer; wash 2, 1% (v/v) Triton X-100 in lysis buffer; wash 3, 1 M NaCl in lysis buffer; wash 4, 5 mM MgCl_2_ and 5 mM ATP in lysis buffer; wash 5, 0.5 M Tris-HCl (pH 7.4) in lysis buffer; wash 6, lysis buffer. The proteins were eluted with 2.5 ml of 50, 100, 200 and 500 mM imidazole and the purest fractions were buffer-exchanged into PBS, using PD-10 columns (VWR, 95017-001). To remove the Trx tag, ∼1.6 mg of each protein was incubated with 25 units of thrombin overnight at room temperature. Ni-NTA beads (70 μl) equilibrated in PBS were added to the mixture for 2 h at room temperature and the samples were centrifuged to remove the His–Trx tag.

For GST–CAML constructs, 1 ml GST Sepharose (GE Healthcare, 17075601) that had been equilibrated in binding buffer (PBS, pH 7.3, 140 mM NaCl, 2.7 mM KCl, 10 mM Na_2_HPO_4_, 1.8 mM KH_2_PO_4_) was added to the filtered supernatant overnight at 4°C. The mixture was added to a poly-prep column and washed with 5 volumes of binding buffer, followed by elution with 0.5 ml of elution buffer (50 mM Tris-HCl, pH 8.0, 20 mM glutathione) and buffer exchange into PBS as described for WRBcc. All proteins were adjusted to a concentration of 100 μM.

### WRBcc binding assay

WRBcc or WRBcc(R77E) (30 μM) was added to 12.5 mg ultralink beads (Thermo Fisher Scientific, 53110) overnight at 4°C, followed by quenching with 10 volumes of 3 M ethanolamine for 2 h at room temperature. The beads were washed in 10 volumes of PBS, 10 volumes of 1 M NaCl and two further washes of 10 volumes of PBS. The supernatant was removed and the 60 μl RRL was added to the beads overnight at 4°C, the unbound supernatant was removed and the beads were analysed for TRC40 binding by immunoblotting.

### SR rescue experiments

Salt-washed rough microsomes (KRMs) were prepared as described previously (Mckenna et al., 2016). Recombinant SR (SRα and SRβ lacking its transmembrane domain, as described in Jadhav et al., 2015) in buffer B2 (50 mM Tris-HCl, pH 8.0, 250 mM NaCl, 10 mM MgCl_2_) was added at a final concentration of 20 nM. For SR rescue assays, SR was added to KRMs at 150 nM, prior to the translation reaction and 15% (v/v) of this mixture was then added to the translation reaction, following puromycin treatment. Controls were KRMs with buffer B2 added, and TKRMs with buffer B2 added. Recombinant SR was a gift from Irmgard Sinning (Heidelberg University, Germany) and was expressed and purified as described previously (Jadhav et al., 2015).

### Statistical analysis

To determine the significance of differences between quantified samples, all samples were compared from ≥3 biological repeat experiments. Cell lysate samples were analysed by immunoblotting and band intensity was determined using ImageStudio software version 2.2.10 (LI-COR Biosciences). Band intensities were first measured and calculated relative to α-tubulin (α-Tub) levels as a loading control and subsequently compared relative to a control sample on the same immunoblot. Statistical analysis of differences between samples and the control was performed using GraphPad Prism 7, as indicated in figure legends. *In vitro* experiments were analysed by phosphorimaging and data was quantified using Aida (Raytek). Statistical analysis was performed in the same way, using GraphPad Prism 7, following sample comparison relative to a negative control sample on the same acrylamide gel.

## Supplementary Material

Supplementary information
