# Supplementary material for: Multiple pathways facilitate the biogenesis of mammalian tail-anchored proteins
Source: J Cell Sci. 2017 Nov 15;130(22):3851–61. doi: 10.1242/jcs.207829 (PMC5702047; doi:10.1242/jcs.207829)
Supplement: Supplementary information [file joces-130-207829-s1.pdf]

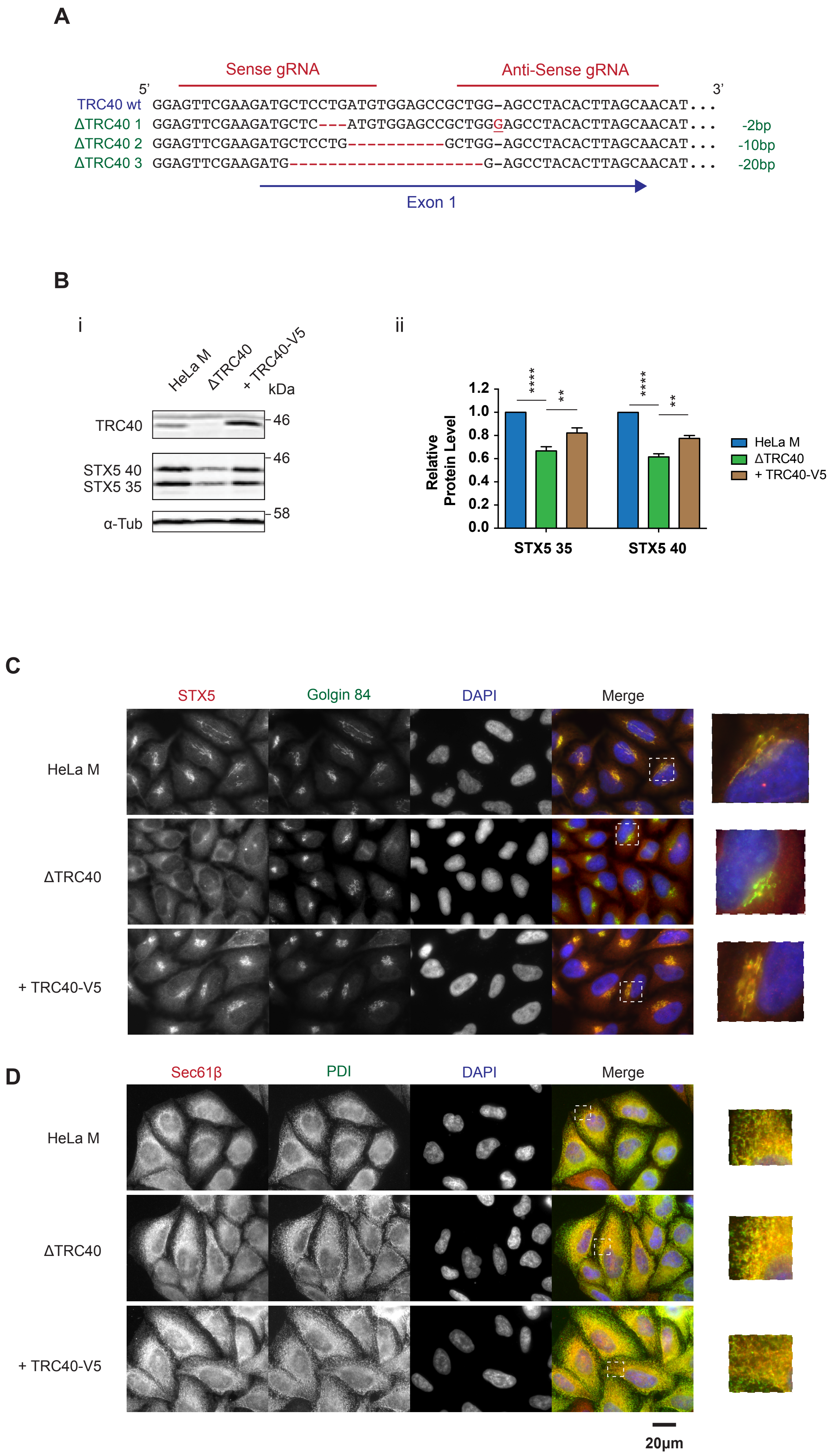

Journal of Cell Science • Supplementary information

Supplementary figure 1 – Deletion of TRC40 has differential effects on endogenous TA protein levels

(A) Sequencing confirmation of the frame shift mutations in genomic TRC40 for each chromosome copy, induced by CRIPSR-Cas9 treatment with gRNAs targeted to TRC40 exon 1. The TRC40 exon 1 binding sites of the gRNAs used to generate the CRISPR knockout HeLa M cell line are shown above the wild-type genomic TRC40 sequence. Frame shift mutations are shown in red, red dashes indicate deletions and underlined red text indicates a nucleotide addition, with the cumulative frame shift in each chromosome sequence shown in green on the right (B) (i) STX5 levels were analysed by immunoblotting extracts from HeLa M cells and ΔTRC40 cells transfected with pcDNA5 as a vector control, and ΔTRC40 cells rescued by TRC40-V5 transfection. (ii) Quantification of TA protein steady state levels from (i) were calculated relative to the HeLa M control. Data shown are mean ±s.d (n=3 biological replicates) and asterisks indicate significance level between samples indicated by a horizontal line. P≤0.01 (**); P≤0.001 (****); (2-way ANOVA). (C-D) Immunofluorescence microscopy images of HeLa M cells, ΔTRC40 cells and ΔTRC40 cells rescued by TRC40-V5 transfection. Cells are stained for the indicated proteins and DAPI (blue). Scale bar = 20 µm, areas of 4x magnification are shown with dashed white box.

Journal of Cell Science • Supplementary information

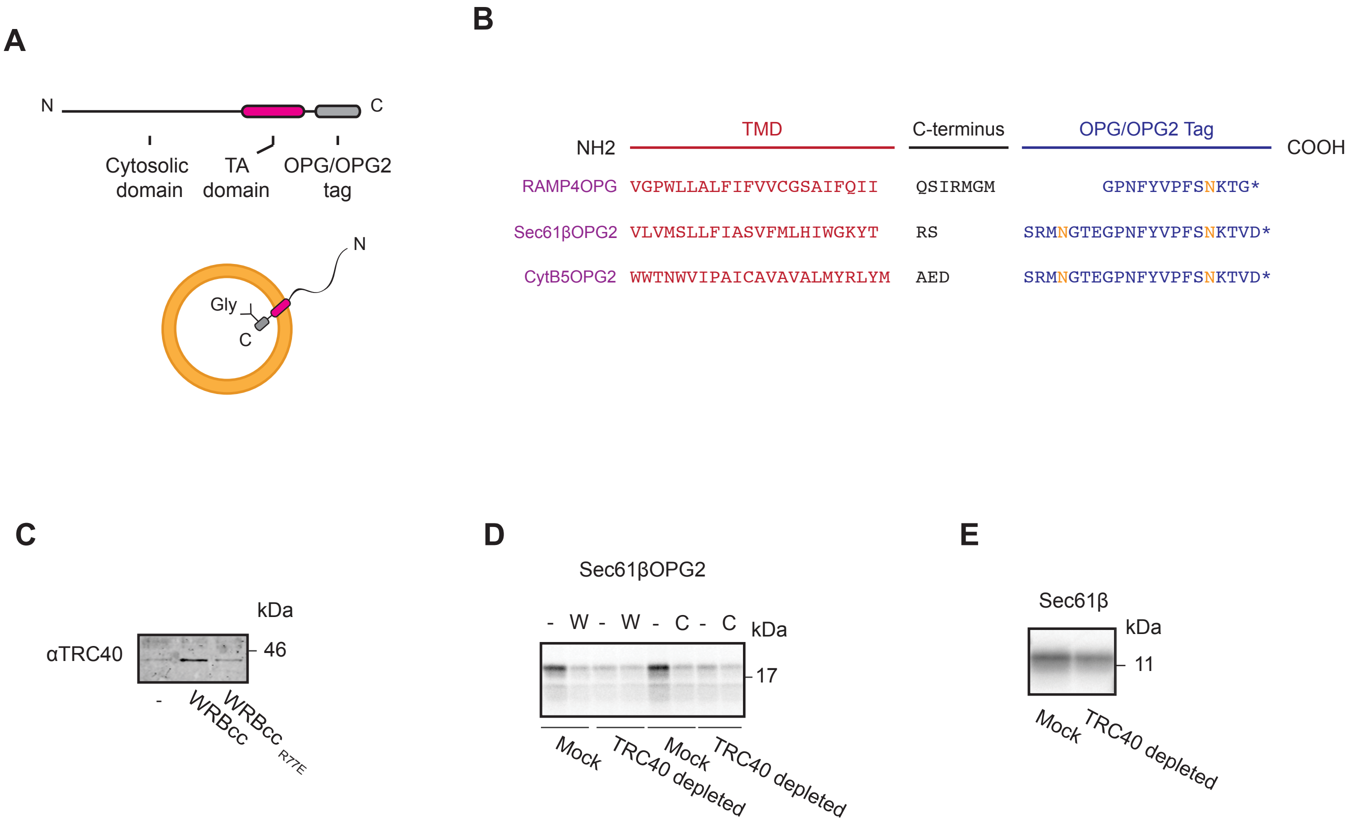

Supplementary figure 2 – TRC40 is not essential for post-translational translocation of TA proteins *in vitro*

(A) Model for the N-glycosylation of OPG-tagged TA proteins, upon insertion into membranes *in vitro*. (B) Sequences of the C-terminal regions of the OPG-tagged TA proteins used in this study, including TMDs and OPG tag. TMD domains are shown in red text, and OPG/OPG2 tag is shown in blue and sequences to the C-terminus of the tag are shown in black. N-glycosylation sites in the OPG/OPG2 tags are shown in orange text. (C) Pull-downs of TRC40 from lysate using immobilised WRBcc or WRBcc(R77E). (D) Phosphorimage of *in vitro* translated Sec61βOPG2 in the presence of lysate or after TRC40 immunodepletion, as well as adding either WRBcc (denoted W) or CAMLcyt (denoted C). (E) Phosphorimage of *in vitro* translated Sec61β after carbonate extraction of membrane-integrated material, in lysate control and after TRC40 depletion.

Journal of Cell Science • Supplementary information

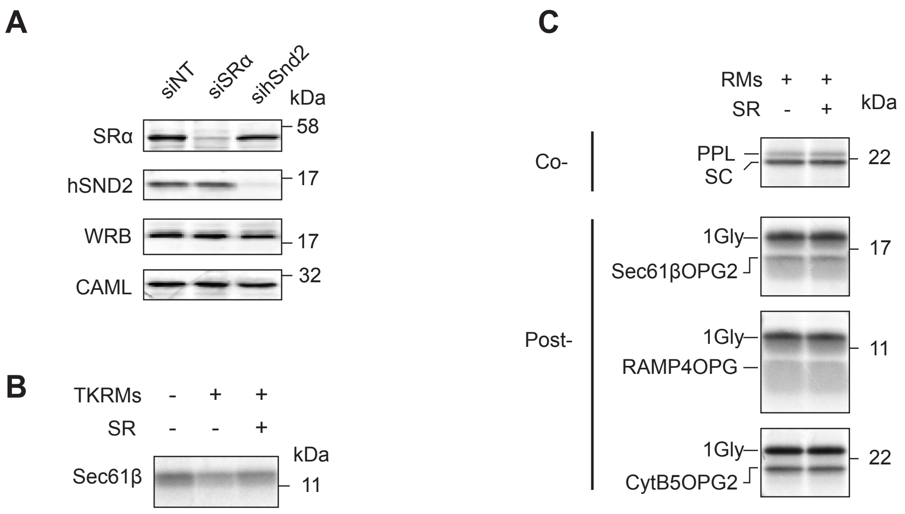

Supplementary figure 3 – The membrane components SRα and hSnd2 promote the biogenesis of TA proteins

(A) HeLa M cells were treated with the indicated siRNAs for 48 h prior to digitonin permeabilisation to deplete the cytosol and enrich for membrane components. The samples were tested for the indicated membrane proteins by immunoblotting. (B) Phosphorimage of the SR rescue experiment, using *in vitro* translated Sec61β lacking the C-terminal OPG tag. (C) Phosphorimage of an SR rescue experiment with PPL, or the indicated TA performed using control non-trypsinised membranes (KRMs) in the absence or presence of recombinant SR, as indicated (cf. Fig 4B in main text).

Journal of Cell Science • Supplementary information

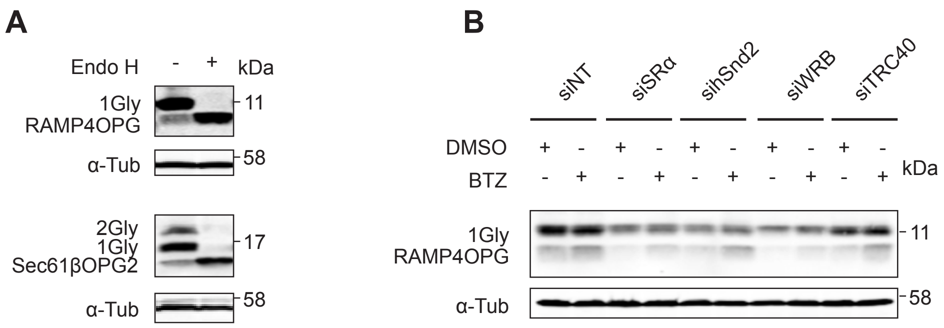

Supplementary figure 4 – Multiple pathways facilitate the ER insertion of tail-anchored proteins

(A) HeLa M cells were transiently transfected with RAMP4OPG or Sec61βOPG2 for 24 h prior to lysate preparation. Samples were either left untreated, or treated with Endoglycosidase H (Endo H) for 1 h, before immunoblotting. (B) HeLa M cells were treated with the indicated siRNAs for 48 h and treated with either DMSO or the proteasome inhibitor, bortezomib (BTZ), for 3 h prior to lysate preparation. Samples were analysed for the proteins indicated by immunoblotting.

Journal of Cell Science • Supplementary information
